# Bacterial EPIYA/EPIYA-like effector motifs and host regulatory pY-SH2 logic: a sequence-informed three-layer convergence model

**DOI:** 10.3389/fcimb.2026.1915865

**Published:** 2026-07-28

**Authors:** Chunyan Wang, Congli Yuan

**Affiliations:** Shanghai Key Laboratory of Veterinary Biotechnology, School of Agriculture and Biology, Shanghai Jiao Tong University, Shanghai, China

**Keywords:** convergent evolution, EPIYA-like motif, ITIM, ITSM, phosphotyrosine, SH2 domain

## Abstract

Bacterial pathogens frequently manipulate host signaling through short linear motifs (SLiMs) that are compact, rapidly evolvable, and often embedded in flexible effector regions. EPIYA/EPIYA-like effector motifs and related bacterial phosphotyrosine (pY) modules are prominent examples because they can be phosphorylated by host tyrosine kinases and subsequently interpreted by host SH2-domain-containing proteins. Here, we develop a sequence-informed and literature-integrated three-layer convergence model in which bacterial EPIYA/EPIYA-like and related pY effector motifs may converge toward the regulatory pY-SH2 logic represented by host ITIM and ITSM motifs. The proposed convergence occurs at three interconnected levels: local Tyr-centered sequence grammar, compatibility with host tyrosine-kinase writing, and SH2-reader recognition. This model does not imply that bacterial motifs are canonical ITIMs or ITSMs, nor does it imply direct homology or functional equivalence. Instead, host ITIM and ITSM motifs are used as reference models for regulatory pY-SH2 docking, whereas bacterial effectors such as CagA, BepD, and Tir illustrate how pathogens can install ectopic pY-centered signaling interfaces. A pLogo-based Tyr-centered comparison provides the sequence-level entry point for this model by visualizing shared Tyr0-centered organization and class-specific flanking-residue patterns among EPIYA/EPIYA-like, ITIM, and ITSM motifs. By shifting the comparison from motif identity to kinase writability and SH2-reader compatibility, this Perspective provides a framework for understanding how bacterial effectors evolve host-readable pY signaling modules.

## 1 Introduction

Pathogenic bacteria often rewire host signaling by encoding SLiMs rather than by reproducing complete host proteins. Such motifs can arise through limited sequence change, are frequently embedded in flexible or disordered regions, and can be positioned within secreted effectors so that host enzymes and modular interaction domains read them after delivery (Backert and Selbach, 2005; Mondino et al., 2020; Sámano-Sánchez and Gibson, 2020; Goswami et al., 2023). Tyrosine-centered bacterial effector motifs are especially informative because their activity depends on two host operations: kinase-mediated writing of phosphotyrosine and domain-mediated reading of that phosphotyrosine (Hunter and Cooper, 1985; Songyang et al., 1994; Hunter, 2009; Roskoski, 2015; Diop et al., 2022).

EPIYA and EPIYA-like motifs are among the clearest examples of this principle. The *Helicobacter pylori* effector CagA is delivered into host cells and phosphorylated on EPIYA segments by host Src-family and Abl-family kinases; phosphorylated CagA then recruits SH2-domain proteins such as SHP2, SHP1 and CSK (Higashi et al., 2002a; b; Tsutsumi et al., 2003; Mueller et al., 2012; Hatakeyama, 2014; Hayashi et al., 2017; Takahashi-Kanemitsu et al., 2020; He et al., 2021). Beyond CagA, tyrosine-phosphorylated bacterial effector motifs have been described across multiple pathogens, including *Bartonella henselae* BepD-F, enteropathogenic *Escherichia coli* Tir, *Chlamydia trachomatis* Tarp, *Anaplasma phagocytophilum* AnkA, *Haemophilus ducreyi* LspA1/LspA2, and the recently reported *Lawsonia intracellularis* LI0666 (Gruenheid et al., 2001; IJdo et al., 2007; Deng et al., 2008; Jewett et al., 2008; Mehlitz et al., 2008; Selbach et al., 2009; Hayashi et al., 2013; Dodd et al., 2014; Sorg et al., 2020; Chen et al., 2022). Although these systems differ in motif sequence, delivery route, kinase usage, reader engagement, and cellular output, they point to a broader principle: bacteria can encode Tyr-centered motifs that become writable by host tyrosine kinases and, in selected cases, readable by host SH2-domain-containing proteins.

The conceptual challenge is to connect these bacterial pY effector motifs with host immune regulatory motifs without collapsing them into the same definition. Host immunoreceptor tyrosine-based inhibitory motifs (ITIMs) and immunoreceptor tyrosine-based switch motifs (ITSMs) are not bacterial EPIYA motifs. They occur in receptor cytoplasmic tails and are embedded in host regulatory networks. However, they are among the best-characterized examples of regulatory pY-SH2 docking logic, in which phosphorylatable tyrosines and flanking residues recruit SH2-domain-containing readers such as SHP1, SHP2, SHIP-family proteins, and SAP-family adaptors (Getahun and Cambier, 2015; Gavrieli et al., 2003; Patsoukis et al., 2020; Marasco et al., 2020; Xu et al., 2021). The central question is therefore not whether bacterial EPIYA-like motifs are ITIMs or ITSMs, but whether these distinct motif classes can be connected through a shared host pY-SH2 signaling logic. We address this question by first comparing Tyr-centered sequence grammar and then integrating published evidence for host kinase phosphorylation and SH2-reader engagement. This sequence-informed and literature-integrated approach leads to a three-layer convergence model.

## 2 Tyr-centered sequence grammar as a basis for comparison

To establish the sequence-level basis for comparison, bacterial EPIYA/EPIYA-like and related pY effector motifs, together with host ITIM and ITSM motifs, were aligned by the central tyrosine residue, defined as Tyr0. The curated motif instances, source annotations, and evidence levels are provided in **Supplementary Table 1**, and the corresponding Y-centered alignments are shown in **Supplementary Figures 1**-**S3** using a −7 to +7 window. For pLogo analysis, a uniform 11-residue window spanning positions −5 to +5 relative to Tyr0 was used to retain the great majority of curated motif occurrences without artificial sequence padding. Motifs lacking a complete native −5/+5 sequence window were retained in **Supplementary Table 1** but excluded from pLogo analysis. Because several bacterial effectors contribute multiple distinct or repeated Tyr-centered motifs, the resulting pLogo patterns are interpreted as descriptive, dataset-dependent visualizations of Tyr0-flanking residue enrichment rather than as population-level estimates of motif prevalence or universal motif rules (O'Shea et al., 2013).

**Figure 1:**
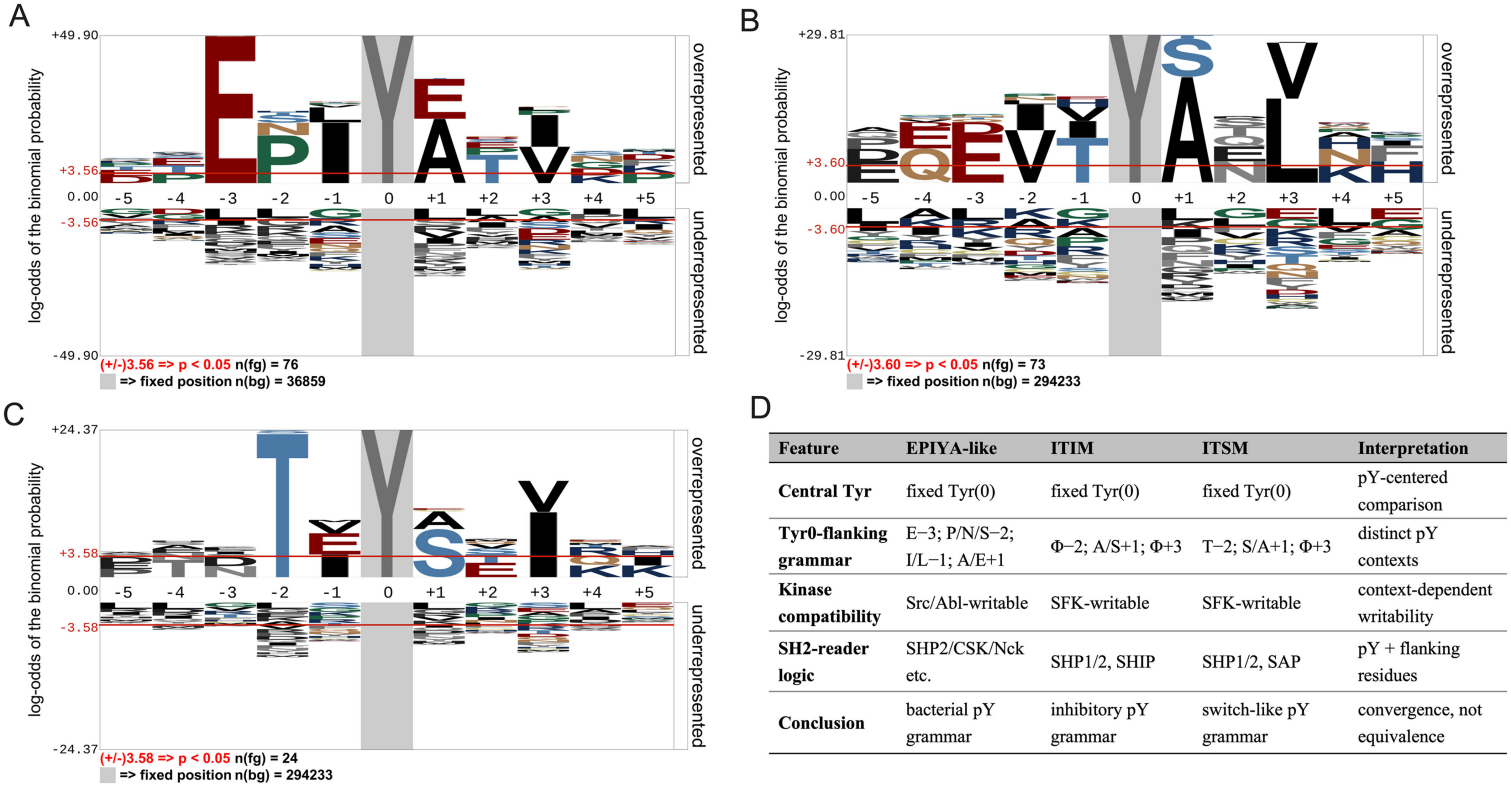
Y-centered sequence grammar comparison of bacterial EPIYA/EPIYA-like motifs and host ITIM/ITSM reference motifs. **(A)** Bacterial EPIYA/EPIYA-like and related pY effector motifs; **(B)** host ITIM motifs; **(C)** host ITSM motifs; and **(D)** concise interpretation of Tyr0-centered sequence features and signaling compatibility. Motifs were aligned by Tyr0 within a −5 to +5 window. Phi (Φ) denotes hydrophobic residues.

This comparison organized the three motif groups within a shared Tyr0-centered coordinate system while highlighting class-specific flanking-residue patterns (**Figure 1**). In the present curated EPIYA-like set, residues corresponding to an EPIYA-centered grammar were recurrent around Tyr0, including enrichment of E at -3, I/L at -1, and A/E at +1, consistent with the canonical EPIYA-centered framing of bacterial EPIYA effectors (Hayashi et al., 2013). In contrast, the ITIM set showed a pattern consistent with canonical inhibitory pY motif organization, including hydrophobic residues around Y-2 and Y + 3, in line with simplified I/V/L/S-x-Y-x-x-L/V consensus-compatible features (Getahun and Cambier, 2015; Avril et al., 2004; Kabat et al., 2002). The ITSM set showed a distinguishable pattern compatible with regulatory or switch-like pY motifs, including recurrent T at -2 and hydrophobic residues around +3, consistent with representative ITSM-containing receptor systems such as PD-1, BTLA, and SLAM-family receptors (Gavrieli et al., 2003; Sidorenko and Clark, 2003; Patsoukis et al., 2020; Xu et al., 2021). Because these patterns depend on motif curation, sample size, and background selection, they are interpreted as dataset-dependent Tyr0-flanking features rather than universal consensus rules.

These observations support the first layer of the convergence model: local sequence grammar convergence around Tyr0. They do not indicate that bacterial EPIYA-like motifs are canonical ITIMs or ITSMs. Rather, they show that bacterial and host regulatory pY motifs can be compared using a common Tyr0-centered coordinate system, and that their flanking-residue environments may shape kinase writability and SH2-reader compatibility. The kinase-compatibility and SH2-reader layers are therefore developed below by integrating this sequence-level comparison with published biochemical and cellular evidence (Songyang et al., 1993; Marasco et al., 2020).

## 3 Host kinase writability of bacterial and regulatory pY motifs

A recurring feature of both host regulatory pY motifs and bacterial EPIYA/EPIYA-like effector motifs is that their signaling potential depends on phosphorylation by host tyrosine kinases. In host immune receptors, ITIM and ITSM phosphorylation is usually mediated by receptor-proximal tyrosine kinases rather than dictated by motif sequence alone. Src-family kinases such as Lck, Fyn, and Lyn contribute to phosphorylation of inhibitory receptor motifs in a receptor-, cell-type-, and activation-state-dependent manner (Latour and Veillette, 2001; Getahun and Cambier, 2015; Shah et al., 2018; Shi et al., 2021). Host kinase writability therefore reflects a combination of local Tyr-centered sequence context, receptor or effector localization, motif accessibility, kinase activation state, and the spatial organization of signaling complexes.

Bacterial EPIYA/EPIYA-like and related pY effector motifs exploit this same principle after delivery into host cells. The CagA paradigm illustrates this most clearly. *Helicobacter pylori* CagA is translocated into host cells and phosphorylated on EPIYA segments by Src-family and Abl-family kinases in a site- and time-dependent manner (Stein et al., 2002; Mueller et al., 2012). Different EPIYA segments are not equivalent substrates: their phosphorylation depends on segment-specific flanking residues, effector localization, and the temporal availability of active host kinases (Higashi et al., 2002a; Mueller et al., 2012; Hayashi et al., 2013). This makes CagA a mature example of bacterial host-kinase writability rather than simply an example of an EPIYA pentapeptide.

The available bacterial examples span different levels of mechanistic resolution. Other effectors broaden this kinase layer beyond canonical CagA-like EPIYA architecture. *Bartonella henselae* BepD contains EPIYA-related motifs that become phosphorylated in host cells and contribute to a phosphorylation-dependent signaling hub associated with STAT3 activation and anti-inflammatory responses (Sorg et al., 2020). BepE and BepF further expand the *Bartonella* repertoire of tyrosine-centered type IV secretion substrates, although motif-specific kinase assignment remains less complete than in the CagA system (Selbach et al., 2009; Hayashi et al., 2013; Wang et al., 2019). Enteropathogenic *Escherichia coli Tir* is not a canonical EPIYA motif, but its Y474 site is phosphorylated after translocation into the host plasma membrane, illustrating that non-EPIYA bacterial pY modules can also become host kinase writable (Kenny, 1999; Gruenheid et al., 2001; Campellone and Leong, 2005). *Chlamydia trachomatis* Tarp similarly undergoes tyrosine phosphorylation during bacterial entry, with Src-family and Abl-family kinases implicated in different experimental settings (Jewett et al., 2008; Mehlitz et al., 2008). *Anaplasma phagocytophilum* AnkA contains EPIYA motifs and is phosphorylated by host kinases including Abl-1 (IJdo et al., 2007; Lin et al., 2007). *Haemophilus ducreyi* LspA1 and LspA2 contain non-canonical EPIYA-like motifs such as EPIYG and EPVYA; tyrosines within these motifs can be phosphorylated by macrophage-encoded protein tyrosine kinases and by purified Src *in vitro* (Deng et al., 2008). The recently described *Lawsonia intracellularis* LI0666 further expands the taxonomic range of bacterial EPIYA effectors, although its endogenous kinase usage and downstream reader engagement remain less fully resolved (Chen et al., 2022).

Together, these observations identify host kinase writability as a second point of comparison between bacterial pY effector motifs and host regulatory pY motifs: both require not only a Tyr-centered sequence context, but also access to the appropriate kinase environment (Latour and Veillette, 2001; Mueller et al., 2012; Getahun and Cambier, 2015).

## 4 SH2-reader compatibility and signaling output

Phosphorylation acquires biological meaning when pY motifs are interpreted by host SH2-domain-containing proteins or SH2-linked signaling complexes. In host immune receptors, phosphorylated ITIM and ITSM motifs convert receptor engagement into signaling output by recruiting readers such as SHP-family phosphatases, SHIP-family inositol phosphatases, or SAP-family adaptors, depending on receptor context and cellular state (Gavrieli et al., 2003; Ott et al., 2001; Getahun and Cambier, 2015; Patsoukis et al., 2020; Xu et al., 2021). SH2-domain recognition is determined by phosphotyrosine and flanking residues, especially positions C-terminal to Tyr0, but also by structural accessibility, avidity, tandem-domain geometry, protein abundance, and competition among readers (Songyang et al., 1993; Marasco et al., 2020; Diop et al., 2022).

Bacterial effectors can install analogous ectopic pY docking sites inside host cells. Phosphorylated CagA recruits SH2-domain-containing proteins including SHP2 and CSK, thereby rewiring epithelial signaling (Higashi et al., 2002b; Tsutsumi et al., 2003; Selbach et al., 2009; Hayashi et al., 2013). SHP2 recruitment by phosphorylated EPIYA-C or EPIYA-D segments is central to CagA-driven morphogenetic signaling, whereas CSK recruitment by phosphorylated EPIYA-A or EPIYA-B segments contributes to feedback regulation of Src-family kinase activity (Higashi et al., 2002a; b; Tsutsumi et al., 2003; Nagase et al., 2015; Hayashi et al., 2017). CagA therefore demonstrates that one bacterial effector can encode multiple phosphorylated reader interfaces, with distinct signaling outputs depending on motif subtype and reader engagement.

Several non-CagA systems support the same reader-layer principle while illustrating different degrees of mechanistic resolution. Phosphorylated Tir Y474 recruits the SH2-containing adaptor Nck, linking a bacterial pY module to N-WASP-Arp2/3-dependent actin pedestal formation (Gruenheid et al., 2001; Campellone and Leong, 2005). AnkA has been reported to recruit SHP1 during early infection, connecting a bacterial EPIYA-containing effector to phosphatase-associated immune modulation (IJdo et al., 2007). BepD uses phosphorylated EPIYA-related motifs as a signaling hub associated with intrinsic STAT3 activation and anti-inflammatory responses; here, STAT3-associated signaling should be interpreted as a pathway output of a phosphorylation-dependent complex rather than as proof that STAT3 is the direct SH2 reader for each BepD motif (Sorg et al., 2020). BepE is particularly informative for the reader layer because phosphorylated BepE EPIYA motifs have been linked to CSK-associated SH2-domain interactions in interactome-level and EPIYA-effector analyses (Selbach et al., 2009; Hayashi et al., 2013; Okujava et al., 2014). LspA proteins also connect EPIYA-like phosphorylation to host signaling modulation, with later studies implicating CSK activation in LspA-mediated inhibition of phagocytosis (Deng et al., 2008; Dodd et al., 2014).

Importantly, these systems do not provide equal levels of evidence: CagA and Tir offer strong motif-to-reader examples, BepD and LspA proteins provide pathway-linked evidence, whereas LI0666 currently represents an emerging EPIYA-effector candidate (Gruenheid et al., 2001; Higashi et al., 2002b; Dodd et al., 2014; Sorg et al., 2020; Chen et al., 2022). Together, these examples identify reader-code compatibility as another point of convergence: selected bacterial pY motifs can be interpreted by the same class of host modular recognition domains that read endogenous regulatory pY motifs (Songyang et al., 1993; Selbach et al., 2009; Marasco et al., 2020).

## 5 A three-layer convergence model

**Figure 2:**
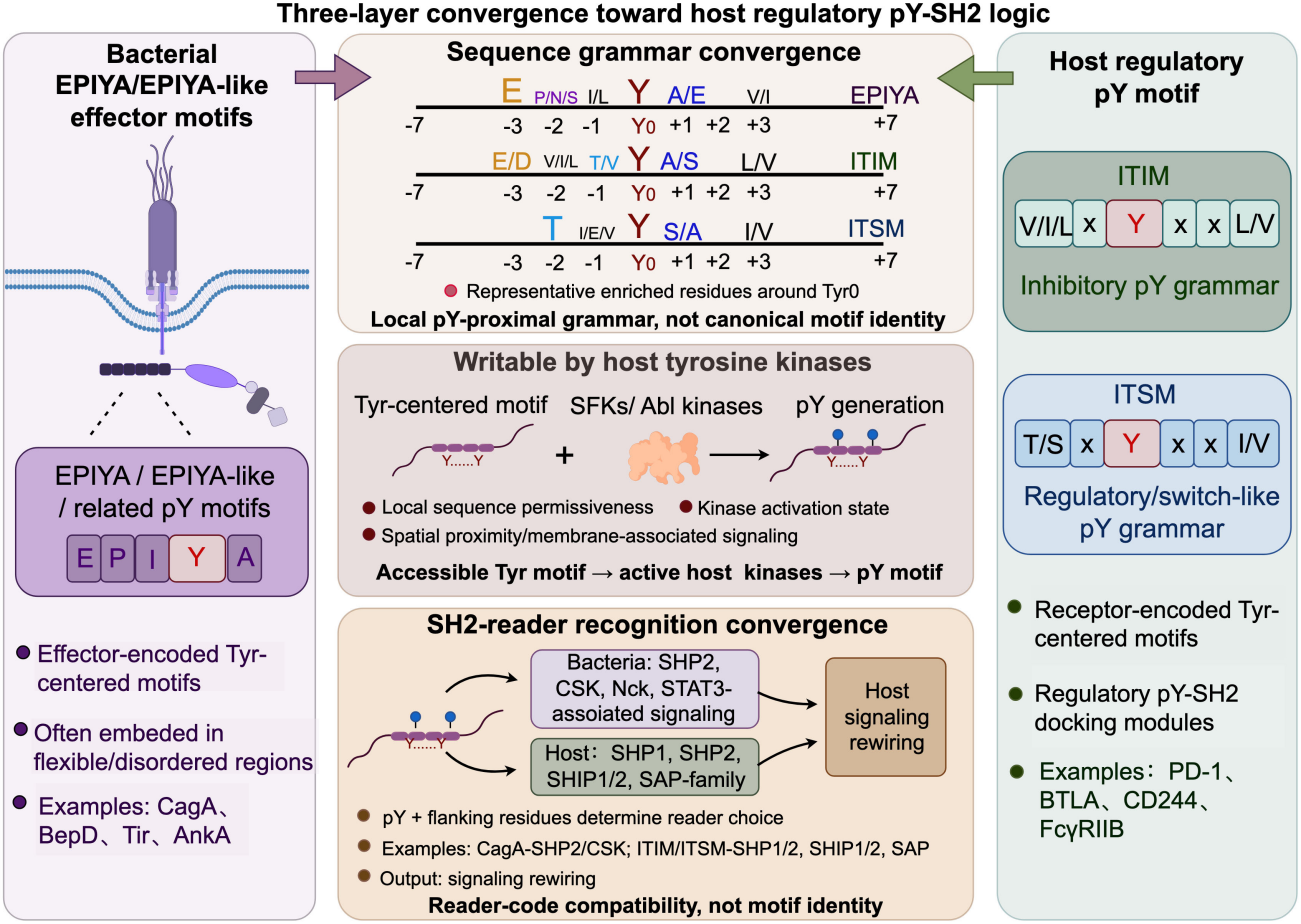
Three-layer convergence model toward host regulatory pY-SH2 logic. The model integrates sequence-level comparison with published mechanistic evidence. Bacterial and host pY motifs are not equivalent motif classes, but they may converge toward a shared biochemical logic comprising: Tyr-centered sequence grammar, host tyrosine-kinase writability, and SH2-reader recognition. The model is a conceptual synthesis, not a claim that all bacterial pY modules engage identical kinases, readers, or outputs. This figure was created using Figdraw 2.0 (Output No. UTTSU62b75).

Taken together, the sequence comparison and mechanistic literature suggest that bacterial EPIYA/EPIYA-like and related pY motifs can be understood through a three-layer convergence model rather than through motif identity alone (**Figure 2**). First, these motifs can be compared with host ITIM and ITSM motifs through local Tyr-centered sequence grammar, as visualized by the Y-centered pLogo analysis (O'Shea et al., 2013). Second, selected bacterial Tyr-centered motifs are writable by host tyrosine kinases in infection-relevant cellular contexts, paralleling the kinase-dependent phosphorylation of host regulatory pY motifs (Stein et al., 2002; Mueller et al., 2012; Getahun and Cambier, 2015). Third, once phosphorylated, some bacterial motifs can be read by SH2-domain-containing proteins or SH2-linked signaling pathways, thereby generating ectopic signaling outputs (Higashi et al., 2002b; Gruenheid et al., 2001; Selbach et al., 2009; Marasco et al., 2020).

This model reframes the relationship between bacterial EPIYA-like motifs and host ITIM/ITSM motifs. It does not propose that bacterial motifs are canonical ITIMs or ITSMs, nor that these motif classes are homologous or functionally equivalent. Rather, ITIM and ITSM motifs serve as reference models for host regulatory pY-SH2 docking logic (Getahun and Cambier, 2015; Patsoukis et al., 2020; Xu et al., 2021). Bacterial effectors appear to exploit the same general signaling architecture - Tyr-centered sequence context, host kinase writing, and SH2-domain reading - to install ectopic pY signaling interfaces during infection (Backert and Selbach, 2005; Hayashi et al., 2013). The value of this model is therefore not to rename bacterial motifs, but to explain how distinct pathogen-encoded tyrosine motifs can enter the same host writing-and-reading circuitry used by endogenous regulatory pY modules.

## 6 Implications and experimental directions

The three-layer convergence model interprets bacterial EPIYA/EPIYA-like and related pY effector motifs beyond a catalogue of phosphorylated sequences. Its central implication is that phylogenetically unrelated effectors, including CagA, BepD, Tir, Tarp, AnkA, LspA proteins, and LI0666, may differ in sequence, delivery route, kinase usage, and output yet converge on a shared signaling logic: Tyr-centered grammar, host tyrosine phosphorylation, and interpretation by SH2-domain proteins or SH2-linked pathways (Hayashi et al., 2013; Sorg et al., 2020; Chen et al., 2022).

The model also defines experimental priorities. Sequence grammar can prioritize motifs in flexible effector regions; infection-relevant phosphorylation should then be tested by phosphoproteomics, targeted mass spectrometry, phospho-specific detection, and Tyr-to-Phe or motif-swap mutagenesis (Higashi et al., 2002a; Gruenheid et al., 2001; IJdo et al., 2007; Deng et al., 2008; Mueller et al., 2012; Sorg et al., 2020). Kinase assignment should combine inhibitors with genetic perturbation, rescue, and *in vitro* kinase assays, because inhibitors alone may reflect indirect effects or kinase redundancy (Jewett et al., 2008; Mehlitz et al., 2008; Mueller et al., 2012). Reader assignment requires phosphopeptide pull-down, SH2-domain arrays, co-immunoprecipitation during infection, quantitative binding assays, structural analysis, and functional rescue to connect individual pY motifs to defined readers and outputs (Selbach et al., 2009; Marasco et al., 2020; Xu et al., 2021).

This framework requires clear boundaries. pLogo-based comparison is a sequence-level organizing analysis, not a direct predictor of kinase specificity or SH2 binding. Bacterial EPIYA-like motifs should not be reclassified as ITIMs or ITSMs, and convergence should not be equated with homology or functional equivalence. Its value is to shift the comparison from motif identity to reader-code compatibility, providing a rigorous framework for discovering and testing host-readable bacterial signaling modules.

## 7 Conclusion

Bacterial EPIYA/EPIYA-like and related pY effector motifs should not be viewed as imperfect copies of host ITIM or ITSM motifs. They are pathogen-encoded Tyr-centered modules that can become compatible with host phosphorylation and SH2-domain reading systems after effector delivery. By integrating motif grammar with evidence for kinase writability, SH2-reader engagement, and signaling output, the three-layer convergence model shifts the focus from motif identity to reader-code compatibility and may help refine how bacterial molecular mimicry is defined.

## Data Availability

The datasets presented in this study can be found in online repositories. The names of the repository/repositories and accession number(s) can be found in the article/**Supplementary Material**.

## Supplementary material

Supplementary Figure 1 Y-centered alignment of curated host ITIM motif instances. Motifs are displayed in a −7 to +7 window centered on Tyr0.

Supplementary Figure 2 Y-centered alignment of curated host ITSM motif instances. Motifs are displayed in a −7 to +7 window centered on Tyr0.

Supplementary Figure 3 Y-centered alignment of curated bacterial EPIYA/EPIYA-like and related pY effector motifs. Motifs are displayed in a −7 to +7 window centered on Tyr0.

Supplementary Table 1 Literature-curated motif source table. The table reports motif class, organism, protein, motif sequence, Tyr0 position where available, evidence level, source reference, and notes on phosphorylation, SH2-reader interaction, or functional evidence where available. All curated motif occurrences are retained, including repeated motifs within individual proteins and identical motifs found in different proteins. Motifs lacking sufficient native flanking sequence to generate a complete −5/+5 window are retained in the table but excluded from pLogo analysis.
